# Emergence of Linezolid Resistance Genes *optrA* and *poxtA* in an Avian *Enterococcus asini*

**DOI:** 10.3390/ijms27093718

**Published:** 2026-04-22

**Authors:** Yuanyuan Li, Huirong Tang, Yating Chen, Yirou Guo, Junhao Hong, Xiao Luo, Jian-Hua Liu, Yi-Yun Liu

**Affiliations:** 1 State Key Laboratory for Animal Disease Control and Prevention, College of Veterinary Medicine, South China Agricultural University, Guangzhou 510642, China; 2 Key Laboratory of Zoonosis of Ministry of Agricultural and Rural Affairs, Guangzhou 510642, China; 3 Guangdong Provincial Key Laboratory of Veterinary Pharmaceutics Development and Safety Evaluation, South China Agricultural University, Guangzhou 510642, China

**Keywords:** linezolid, an oxazolidinone antibiotic of last resort, multidrug-resistant (MDR) Gram-positive bacteria, *optrA*, *poxtA*, plasmids, *Enterococcus asini*

## Abstract

Linezolid represents a critical last-resort treatment for severe multidrug-resistant (MDR) Gram-positive bacterial infections. Rising linezolid resistance in *Enterococcus* isolates threatens its efficacy; this study characterized the molecular features and transfer potential of plasmid-encoded linezolid resistance genes *optrA* and *poxtA* in a linezolid-resistant *Enterococcus asini* isolate from chickens. An *E. asini* strain was isolated during a surveillance program focusing on drug-resistant Gram-positive bacteria in poultry. PCR screened linezolid resistance genes, conjugation and plasmid stability assays evaluated gene transferability and stability, and whole-genome sequencing (WGS) was performed using both the Illumina and Nanopore platforms. We present the first detection of *optrA* and *poxtA* genes in *E. asini* recovered from chicken feces in China. Sequence analysis of the complete genome showed that *poxtA* and *optrA* were situated on two distinct plasmids. The *poxtA* positive plasmid, pHNGXN23C145Ea-1, also carried multiple resistance genes, including *tet(S)*, *fexB*, *erm(B)*, *ant(6)-Ia*, *aph(3′)-III*. Furthermore, the *poxtA* gene was flanked by IS*1216E* mobile elements. The *optrA* bearing plasmid, pHNGXN23C145Ea-2, harbours a common genetic array of ‘IS*1216E fexA-optrA-erm(A)*-IS*1216E*’. Conjugation experiments indicated that neither the *poxtA*- nor the *optrA*-bearing plasmid was transferred to recipient strains, which was consistent with sequence analysis showing that both plasmids lacked intact conjugative transfer regions. Stability assays confirmed that *poxtA* and *optrA* remained highly stable in the absence of selective pressure. Notably, this discovery was made in a livestock sample, despite the non-use of linezolid in food animals, suggesting that such niches may act as silent reservoirs for resistance genes, which could persist and potentially transfer to clinically relevant MDR pathogens.

## 1. Introduction

Linezolid, a last-resort oxazolidinone antibiotic, is critical for the management of severe infections caused by multidrug-resistant (MDR) Gram-positive pathogens, including methicillin-resistant *Staphylococcus aureus* (MRSA) and vancomycin-resistant enterococci (VRE), as well as *Streptococcus pneumoniae*. While oxazolidinones are not authorized for veterinary use in food-producing animals, three acquired resistance genes (*cfr*, *optrA*, and *poxtA*) that confer cross-resistance to oxazolidinones and phenicols have been increasingly identified in bacteria isolated from animal sources [1]. This growing trend raises substantial concerns regarding the risk of zoonotic transmission and environmental dissemination [2].

Linezolid resistance in *Enterococcus* is governed by three primary mechanisms: (i) point mutations arising within the central loop of domain V of the 23S rRNA (most frequently G2576T, alongside G2447T and G2505A), which disrupt drug interaction at the peptidyl transferase center [3]; (ii) acquisition of horizontally transferable resistance determinants, encompassing *cfr* and its allelic variants, as well as the ABC-F protein genes *optrA* and *poxtA* [4]; and (iii) amino acid substitutions in ribosomal proteins L3, L4, and L22, encoded by *rplC*, *rplD*, and *rplV* respectively, which further diminish susceptibility profiles [5]. Within this framework, the *cfr* gene family encodes 23S rRNA methyltransferases that impart the PhLOPSA multidrug resistance phenotype, conferring resistance to phenicols, lincosamides, oxazolidinones, pleuromutilins, and streptogramin A [6]. Conversely, both *optrA* and *poxtA*, members of the ABC-F protein superfamily, exert their resistance via ribosomal protection mechanisms, resulting in reduced susceptibility to oxazolidinones (including tedizolid) and phenicols; additionally, *poxtA* has been associated with diminished susceptibility to specific tetracycline derivatives [4].

Among these acquired determinants, *optrA* and *poxtA* have emerged as the most frequently reported and rapidly disseminating linezolid resistance genes in recent years [7,8]. Since its initial discovery in enterococci in 2015, *optrA* has been detected across numerous countries, with particularly high prevalence in China [9]. The *poxtA* gene was first characterized in a clinical methicillin-resistant *Staphylococcus aureus* (MRSA) isolate in 2018 and has since been reported in *Enterococcus* spp. and *Lactobacillus* spp. from human, animal, and environmental sources, with enterococci being the predominant host to date [10,11]. A mounting public health concern is the co-occurrence of multiple linezolid resistance determinants within a single bacterial isolate, occasionally localized on the same plasmid, which may facilitate horizontal gene transfer and accelerate their dissemination [12]. Despite these insights, significant knowledge gaps persist regarding the host range and plasmid ecology of linezolid resistance genes, particularly among less studied enterococcal species and within avian production systems.

Here, we report, to our knowledge, the first identification of an avian *Enterococcus asini* isolate (GXN23C145Ea) co-harboring *optrA* and *poxtA*. Using short- and long-read WGS, alongside conjugation and stability assays, we resolve the mobile genetic elements (insertion sequences, transposons, and plasmid backbones) and infer the molecular mechanisms and transmission characteristics underlying antimicrobial-resistance gene dissemination. These findings contribute to the risk assessment and development of control strategies targeting animal-derived enterococci and their transferable linezolid-resistance reservoirs.

## 2. Results and Discussion

The *Enterococcus asini* isolate GXN23C145Ea was recovered from chicken feces as part of a 2023 surveillance initiative targeting oxazolidinone-nonsusceptible Gram-positive bacteria. Antimicrobial susceptibility profiling demonstrated a multidrug-resistant phenotype, with the strain showing resistance to linezolid, gentamicin, tetracycline, florfenicol, and erythromycin, while retaining susceptibility to enrofloxacin and vancomycin (Table 1). Consistent with the detected resistance determinants, *PoxtA* has been reported to confer variable cross-effects on tetracyclines [4]. In this study, the high-level tetracycline resistance is most likely driven by the presence of *tet(S)*, while *poxtA* may additionally contribute to this phenotype, suggesting a combined effect of these resistance determinants. Notably, it demonstrated resistance not only to linezolid but also high-level resistance to the second-generation oxazolidinone tedizolid, which is clinically concerning given that tedizolid is considered a last-resort option for multidrug-resistant Gram-positive infections [13]. This profile, particularly the co-resistance to both oxazolidinone generations, suggests the acquisition of robust ribosomal protection and/or associated accessory resistance mechanisms [14]. Reports of *E. asini* remain scarce since its first description from donkey cecum in 1998 [15], and it is primarily regarded as a commensal organism of the animal gastrointestinal tract [16]. To date, there are no well-documented reports of its isolation from human clinical samples. Nevertheless, as a member of the enterococcal community, it occupies ecological niches where horizontal gene transfer is frequent and may serve as a reservoir for antimicrobial resistance genes. To our knowledge, this study provides the first documentation of the co-occurrence of *optrA* and *poxtA* in this species. This finding significantly expands the recognized resistome of this neglected enterococcal species and further implicates it as a potential reservoir for oxazolidinone resistance.

Whole-genome sequencing analysis demonstrated that strain GXN23C145Ea carried a diverse array of antimicrobial resistance determinants, encompassing the aminoglycoside resistance genes *aac(6′)-aph(2″)*, *ant(6)-Ia* and *ant(6)-Ia/aph(3′)-III*, the tetracycline resistance gene *tet(S)*, the trimethoprim resistance gene *dfrG*, the macrolide resistance genes *erm(B)* and *erm(A)*, the chloramphenicol and florfenicol resistance gene *fexA*, and the oxazolidinone resistance genes *optrA* and *poxtA*. Mechanistically, OptrA and PoxtA are ABC-F ribosomal protection proteins that reduce oxazolidinone binding at the peptidyl-transferase centre and commonly coexist with phenicol (fexA/B) and macrolide genes on shared mobile modules [14,17], enabling elevated MICs to linezolid/tedizolid and cross-effects on phenicols (and variably, tetracyclines for PoxtA). These features account for the antimicrobial susceptibility phenotype observed in antimicrobial susceptibility (Table 1).

**Figure 1:**
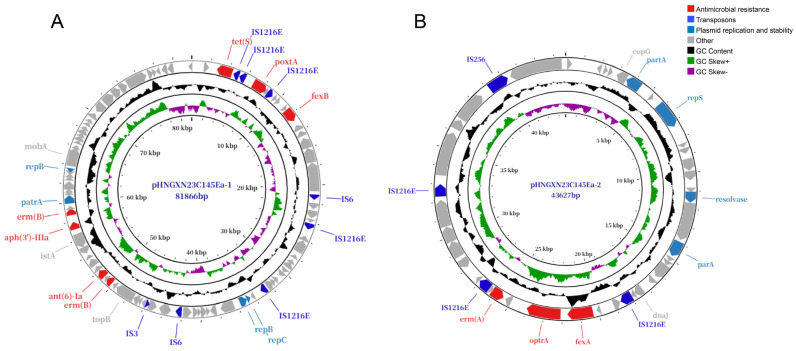
Schematic maps of the *poxtA*-harboring plasmid pHNGXN23C145Ea-1 (**A**) and the *optrA*-carrying plasmid pHNGXN23C145Ea-2 (**B**) from *E. asini* strain GXN23C145Ea. The concentric rings represent (from outer to inner) (i) the locations of predicted open reading frames (ORFs) transcribed in the counterclockwise direction; (ii) the GC content profile (shown as the black ring, with variable segments indicating relative GC levels along the plasmid sequence); (iii) GC skew [(G − C)/(G + C)] (green ring: positive skew; dark purple ring: negative skew); and (iv) the size scale in base pairs (bp). Genes are color-coded according to their functional annotations.

WGS revealed a total genome size of roughly 2.62 Mb, which consisted of a 2,486,628-bp chromosomal scaffold and three distinct plasmids, designated pHNGXN23C145Ea-1, pHNGXN23C145Ea-2 and pHNGXN23C145Ea-3. Gene localization analysis revealed that the chromosome carried the aminoglycoside resistance gene *aac(6′)-aph(2″)* and the trimethoprim resistance gene *dfrG*. Meanwhile, *poxtA* was carried by plasmid pHNGXN23C145Ea-1 together with *tet(S)*, *fexB*, *erm(B)*, *ant(6)-Ia* and *aph(3′)-III*, whereas *optrA* was located on plasmid pHNGXN23C145Ea-2 together with *erm(A)* and *fexA*. Untyped plasmid pHNGXN23C145Ea-3 contained no detectable resistance genes (Figure 1). Notably, although a relaxase gene (*mobA*) was identified on the *poxtA*-bearing plasmid, no recognizable origin of transfer (*oriT*) sequence was detected, suggesting an incomplete mobilization system and a low likelihood of helper-mediated transfer. In line with these genotypic findings, in silico sequence analysis verified that both plasmids lacked intact conjugative transfer modules, which accounts for their inability to undergo horizontal transfer. Consistent with this, conjugation experiments demonstrated that neither the *poxtA*-carrying nor the *optrA*-harboring plasmid was successfully transferred to recipient strains. However, the *optrA* gene was bracketed by two IS*1216E* elements oriented in the same direction. Subsequent inverse PCR confirmed the formation of a circular IS*1216E*-mediated intermediate, suggesting an alternative, potential mechanism for the mobilization of the *optrA* cassette despite the non-conjugative plasmid backbone [18].

**Figure 2:**
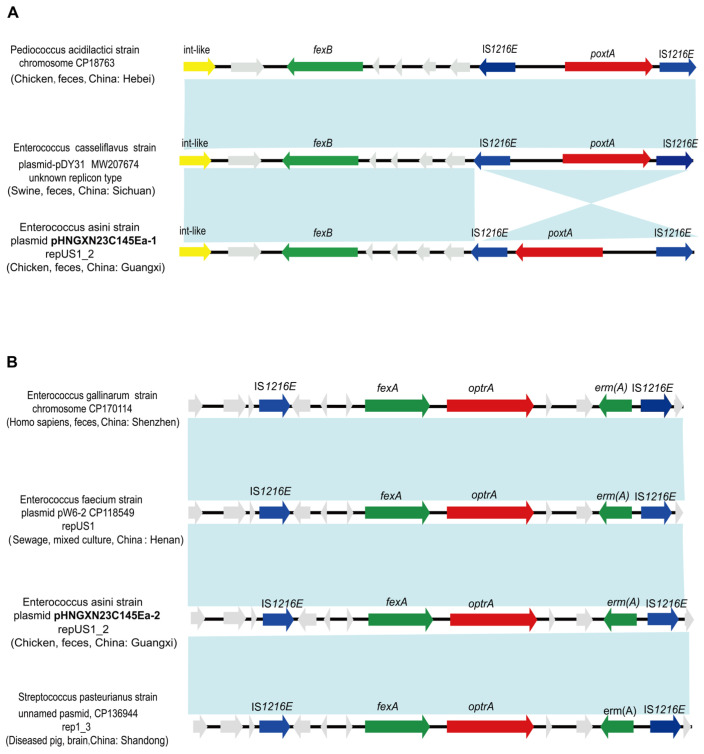
Comparison of *optrA*/*poxtA*-co-carrying plasmids and their genetic contexts. (**A**) Comparison of pHNGX23C145Ea-1 (harboring *poxtA*) with structurally similar plasmids isolated from different strains and sources. (**B**) Genetic context comparison of plasmid pHNGX23C145Ea-2 (carrying *optrA*) with homologous genetic elements from chromosomes/plasmids of distinct strains. Genes with distinct functional roles are represented by arrows of different colors. Resistance genes, mobile elements, putative tyrosine recombinases, and other genomic features are color-coded green, blue, yellow, and gray, respectively. Regions of sequence homology (nucleotide identity ≥ 99%) are indicated by light blue shading.

To investigate the genetic context of the identified resistance plasmids, BLASTn analysis was performed against the GenBank database. This revealed that pHNGXN23C145Ea-1 harbored a highly conserved *poxtA*-containing region with the genetic organization *tyr*-like-*bcrR*-*fexB*-hp1-hp2-IS*1216E*-*poxtA*-IS*1216E*. This region exhibited high sequence similarity to corresponding loci located on the chromosome of *Pediococcus acidilactici* and the plasmid pDY31 from *Enterococcus casseliflavus* (Figure 2A). As emphasized in previous studies [18], this conserved genetic architecture, in which *poxtA* and *fexB* coexist and are flanked by functional IS*1216E* elements, facilitates horizontal transfer of *poxtA* via IS*1216E*-mediated transposition or plasmid fusion, while *fexB* confers florfenicol resistance and provides coselection pressure that promotes the persistence of *poxtA* even in the absence of oxazolidinone exposure. In addition, the consistent presence of an upstream putative tyrosine recombinase family protein suggests that site-specific recombination may further contribute to the excision, integration, or stabilization of the IS*1216E*-associated *poxtA* region [19], thereby enhancing its dissemination across different bacterial hosts.

BLASTn analysis showed that plasmid pHNGXN23C145Ea-2 shared high overall sequence similarity with sequences from diverse sources in the GenBank database, with >40% coverage and >99.9% identity. These included plasmid pW6-2 from *Enterococcus faecium* (40% coverage, 99.99% identity, CP118549, environmental source, Henan Province, China), an unnamed plasmid from *Streptococcus pasteurianus* (43% coverage, 99.99% identity, CP136944, swine origin, Hainan Province, China), and a chromosomal sequence from *Enterococcus gallinarum* (41% coverage, 99.95% identity, CP170114, human fecal origin, Shenzhen, China). The *optrA*-carrying plasmid pHNGXN23C145Ea-2 belongs to the repUS41_1 replicon family, whereas plasmid pW6-2 (CP118549) is classified as repUS1_2 and the unnamed plasmid (CP136944) as rep1_3, indicating that these elements are associated with diverse plasmid backbones. Comparative genomic analysis revealed that all these elements harbor an identical resistance module—*fexA-optrA-erm(A)*—flanked by IS*1216E* insertion sequences, potentially forming a composite transposon-like structure (Figure 2B). Despite this conserved module, the surrounding genetic backbones differ substantially, suggesting that only the resistance cassette, rather than the entire plasmid, is shared among these elements. Inverse PCR confirmed that the two IS*1216E* elements can form a circular intermediate, indicating that IS*1216E*-mediated recombination is a key mechanism for the horizontal transfer of this *optrA*-containing cassette, consistent with previous reports [18]. However, the frequency of circular intermediate formation was not determined, and its contribution to gene dissemination under natural conditions remains to be further investigated.

The stability of both plasmids, pHNGXN23C145Ea-1 (*poxtA*) and pHNGXN23C145Ea-2 (*optrA*), was assessed through serial passage in antibiotic-free BHI broth. Both exhibited 100% retention after approximately 7 days, demonstrating high in-host stability even in the absence of selective pressure.

In summary, the *optrA* and *poxtA* genes in *E. asini* GXN23C145Ea were located on two distinct, stable plasmids. While the *poxtA*-bearing plasmid was non-mobilizable, the *optrA*-carrying plasmid (pHNGXN23C145Ea-2) possesses a mobilizable genetic architecture. The high sequence homology of pHNGXN23C145Ea-2 with genetic elements from *Enterococcus* and *Streptococcus* species across human, animal, and environmental reservoirs in different geographical regions indicates that this *optrA-erm(A)-fexA* platform is capable of interspecies and cross-sectoral transmission. The co-localization of resistance genes to oxazolidinones, phenicols, and macrolides on a single, mobilizable unit poses a significant public health risk, facilitating the dissemination of multi-drug resistance among Gram-positive pathogens.

## 3. Materials and Methods

### 3.1. Bacterial Strains and PCR Analysis

As part of a 2023 surveillance initiative focused on drug-resistant Gram-positive bacteria in poultry, the *Enterococcus asini* strain GXN23C145Ea was isolated from a poultry farm situated in Nanning, Guangxi, China. Species-level identification of the bacterial isolate was performed via MALDI-TOF MS (Bruker Daltonik GmbH, Bremen, Germany). To verify the presence of oxazolidinone resistance genes and their flanking regions, PCR amplification followed by Sanger sequencing was conducted with the primers detailed in Appendix A, Table A1 to confirm the genetic context inferred from whole-genome sequencing data.

### 3.2. Antimicrobial Susceptibility Testing

The antimicrobial susceptibility of *Enterococcus asini* strain GXN23C145Ea was assessed using broth microdilution assays, following the guidelines set by the Clinical and Laboratory Standards Institute (CLSI) [20]. *E. faecalis* ATCC 29212 and *Staphylococcus aureus* ATCC 29213 served as quality control strains. The panel of antimicrobials evaluated in this study included gentamicin, erythromycin, tetracycline, florfenicol, enrofloxacin, linezolid, tedizolid, and vancomycin. Interpretation of susceptibility results was performed in accordance with the latest CLSI clinical breakpoints.

### 3.3. WGS and Sequence Analysis

Genomic DNA was isolated from *Enterococcus asini* strain GXN23C145Ea using the HiPure Bacterial DNA Kit (Magen, Beijing, China) following the manufacturer’s protocol. Whole-genome sequencing was conducted on the Illumina HiSeq X Ten platform (Illumina, Inc., San Diego, CA, USA) and the QitanTech nanopore sequencing system (QitanTech, Chengdu, China). Hybrid de novo assembly of the combined short- and long-read datasets was carried out with Unicycler v0.4.7 [21]. Antimicrobial resistance genes and plasmid replicons were identified and typed using the bioinformatics tool ABRicate v0.8 (https://github.com/tseemann/abricate, accessed on 19 September 2025). In addition, open reading frames (ORFs) in the assembled genome sequences were annotated via the RAST server (https://rast.nmpdr.org/, accessed on 19 September 2025), a rapid online annotation platform. Comparative sequence analysis of plasmid sequences was performed using BLASTN against the NCBI nucleotide database (https://blast.ncbi.nlm.nih.gov/Blast.cgi, accessed on 21 September 2025).

### 3.4. Conjugation Experiments

To assess the horizontal transfer capacity of the *optrA* and *poxtA* resistance genes, the *E. asini* strain was employed as the donor in conjugation assays, with rifampicin-resistant *E. faecalis* JH2-2 as the recipient. Donor and recipient cultures were combined at a 1:1 ratio before the mating process. Following incubation at 37 °C for 16–24 h, colonies growing on selective agar plates supplemented with 100 mg/L rifampin and 10 mg/L florfenicol were identified as potential transconjugants. These transconjugants were confirmed to the species level by MALDI-TOF MS, and the presence of the target resistance genes was detected via PCR.

### 3.5. Plasmid Stability Testing

To assess the stability of the plasmids carrying the linezolid resistance genes *optrA* and *poxtA*, the *Enterococcus asini* strain GXN23C145Ea was evaluated in a plasmid stability assay using antibiotic-free BHI broth. Three independent single colonies of the *optrA*- and *poxtA*-positive isolate were incubated overnight at 37 °C in 3 mL of antibiotic-free BHI medium. Thereafter, 3 μL of the overnight culture was added to 3 mL of fresh BHI broth, diluted 1000 times, and then subcultured for 7 d. On each day of the experiment, 100 colonies were randomly picked, and the presence of the *optrA* and *poxtA* resistance genes was verified via PCR amplification. The plasmid retention rate was determined as the proportion of colonies positive for both *optrA* and *poxtA* relative to the total 100 colonies screened.

### 3.6. Accession Numbers

The whole-genome sequence of *E. asini* strain GXN23C145Ea has been deposited in the GenBank database under the BioProject accession number PRJNA1404451.

## 4. Conclusions

To the best of our knowledge, this study presents the first documented case of a linezolid-resistant *Enterococcus asini* isolate co-harboring *optrA* and *poxtA* recovered from a poultry origin. Our findings significantly expand the known resistance gene pool of this understudied species and underscore its potential role as a “hidden reservoir” that facilitates the dissemination of oxazolidinone resistance. The identification of a widely disseminated, IS*1216E*-mobilizable *optrA* platform across multiple bacterial genera and reservoirs (human, animal, environmental) raises significant public health concerns. The detection of such strains in food-producing animals necessitates ongoing surveillance to monitor the transmission of these critical resistance determinants.

## Figures and Tables

**Table 1:** Characterization of GXN23C145Ea.

Strain	Location	Size (bp)	Plasmid Type	Drug Resistance Genes	MIC(μg/mL)							
					GEN	ERY	TET	FLOR	ENR	VAN	LIN	TED
GXN23C 145Ea	chromosome	2,486,628	/	*aac(6′)-aph(2* *″* *)/dfrG*	128	128	>128	128	2	1	8	<128
	pHNGXN23C 145Ea-1	81,866	repUS1_2_rep(pVEF3)	*tet(S)/poxtA/fexB/erm(B)/ant(6)-Ia/aph(3′)-III*								
	pHNGXN23C 145Ea-2	43,627	repUS41_1_repB(PML21)	*fexA/optrA/erm(A)*								
	pHNGXN23C 145Ea-3	8467	/									

GEN, gentamicin; ERY, erythromycin; TET, tetracycline; FLOR, florfenicol; ENR, enrofloxacin; VAN, vancomycin; LIN, linezolid; TED, tedizolid.

## Data Availability

The original contributions presented in this study are included in the article. Further inquiries can be directed to the corresponding author.

## Appendix A

**Table A1:** PCR primers used in this study.

Target	Primer	Sequence (5′-3′)	Product Size (bp)	Reference or Source
*optrA*	*optrA*-F	CAGGTGGTCAGCGAACTAAGA	792	This study
	*optrA*-R	AGCCAAGAGCAGTTCTGACC		
*poxtA*	*poxtA*-F	GGAAGTTGCTCAGTACGGCT	975	This study
	*poxtA*-R	TCAATGCAGAGCAGGAAGCA		
*optrA* minicircle in plasmid pHNGXN23C145Ea-2	Circ-*optrA*-F	TGCACATACTTGAAACCTCC	5230	This study
	Circ-*optrA*-R	CTTGAACTACTGATTCTCGG		
